# Performance of Holstein calves receiving equal solids per day from high-solids milk and milk replacer blend once or twice daily combined with dry versus wet fine-particle starter diet

**DOI:** 10.3168/jdsc.2021-0194

**Published:** 2022-07-09

**Authors:** H. Beiranvand, F. Ahmadi, R. Tahmasbi, M.R. Farokhzad, M. Ariana, M.H. Ghaffari

**Affiliations:** 1 Chaltasian Agri.-Animal Production Complex, Varamin, Tehran, 33751-13111, Iran; 2 Department of Eco-Friendly Livestock Science, Institute of Green Bio Science and Technology, Seoul National University, Pyeongchang 25354, Gangwon-do, Korea; 3 Department of Animal Science, Faculty of Agriculture, Shahid Bahonar University of Kerman, Kerman, Iran; 4 Alltech Lienert Australia, 8 Roseworthy Road, Roseworthy SA 5371, Australia; 5 Department of Animal Science, Khorramabad Branch, Islamic Azad University, Khorramabad 68178-16645, Iran; 6 Institute of Animal Science, University of Bonn, 53115 Bonn, Germany

## Abstract

This study examined the effects of frequency of feeding high-solids milk and milk replacer (MMR) blend combined with dry matter (DM) content of starter feed on the performance of dairy calves. Forty-eight Holstein calves (40.2 ± 3.33 kg of body weight) were randomly assigned to 1 of 4 treatments in a completely randomized design. Based on a combination of 2 MMR feeding frequencies (once versus twice daily) and 2 starter DM levels (91.1 and 50% DM), 4 treatments were designed as (1) once daily + dry starter feed (OD), (2) once daily + wet starter feed (OW), (3) twice daily + dry starter feed (TD), and (4) twice daily + wet starter feed (TW). The amount of milk replacer powder added to whole milk was adjusted at each feeding frequency to ensure that calves received similar solids from the MMR blend via the twice-daily (total solids = 18.6%) or once-daily (total solids = 31.0%) programs. Starter intake in the preweaning period did not differ between treatments (average 0.39 kg of DM/d), but the postweaning starter intake was greater in calves fed the wet starter (OW and TW) than in those fed the dry starter (OD and TD). Calves receiving the high-solids MMR blend twice versus once daily grew faster before weaning, which was reflected by a greater weaning weight in the TD and TW groups (88.9 kg) than in the OD and OW groups (83.2 kg). The daily growth rate after weaning did not differ between treatments and averaged 1.15 kg/d. Gain-to-feed ratio during the preweaning period was greater in calves from TD and TW (mean 0.67) than in calves from the OD or OW groups (mean 0.58). Postweaning gain-to-feed ratio was not different among treatments and averaged 0.38. Days to first diarrhea and duration of diarrhea (4.66 ± 0.38), as well as days that animals were treated with antibiotics, remained unaffected with treatments. Although preweaning body weight gain was lower with once-daily milk feeding, calves were twice as heavy at weaning as at birth in both milk feeding programs regardless of starter moisture content, which may qualify the once-daily milk feeding program as a cost-effective management strategy in commercial calf-rearing operations. Feeding solutions with a high solids content (above 15%) may be associated with an increased risk of abomasal bloat and should be used with caution.

## Highlights

• Feeding a high-solids milk replacer blend once versus twice per day reduced average daily gain before weaning.
• Both liquid feeding programs doubled calf birth weight by weaning.
• Addition of water to dry calf starter resulted in an increase in starter DMI after weaning.

## Graphical Abstract

**Figure:**
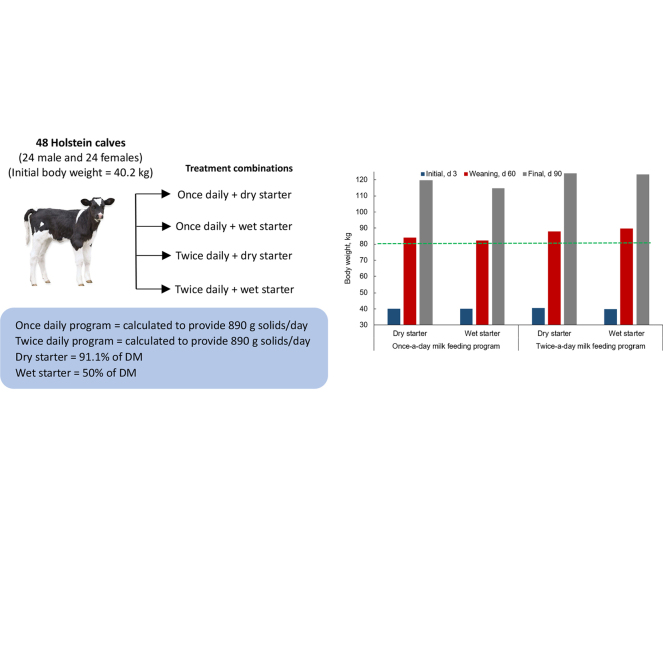
**Summary:** Feeding a similar amount of nutrients via a once-daily versus a twice-daily feeding program based on a blend of milk and milk replacer resulted in a reduction in calf growth rate before weaning, but also doubled calf birth weight by weaning. The addition of water to dry fine-particle starter feed increased the starter intake only after weaning. Feeding solutions with a high osmolality, as used in a once-daily program, should be used with caution because they may increase the risk of abomasal bloat..

Management and feeding practices that maximize nutrient intake from both liquid and solid diets can improve calf health and performance. Feeding milk once daily may be a management strategy that directly contributes to labor savings, the largest cost factor in commercial calf-rearing operations (Hulbert et al., 2011), and could be particularly important in emergency situations such as the COVID-19 pandemic that resulted in a shortage of labor in livestock production systems. Previous studies found negligible difference in growth performance and health of calves raised on feeding programs that offered whole milk once or twice daily (Kehoe et al., 2007; Saldana et al., 2019). In addition, Kienitz et al. (2017) found slight differences in growth rate and body measurements development in calves fed milk once and twice daily, resulting in lower labor costs and thus increased economic returns. In contrast to these studies using a high volume of milk (6 L or more/d; TS = 13%), less is known about how newborn calves develop when offered the same concentration of solids per day from a high-solids milk and milk replacer (**MMR**) blend (whole milk + milk replacer powder) once versus twice daily.

Our previous studies have shown that feeding young calves a wet starter feed in summer (50% DM) and winter (75% DM) resulted in significant increases in solid feed intake and growth efficiency (Beiranvand et al., 2016, 2019). Increased adhesion of starter feed particles by the addition of water allowed calves to consume more fine particles, which was associated with increased nutrient and energy consumption and promoted calf growth and the development of more functional rumen fermentation (Beiranvand et al., 2019).

Previous studies reported the comparative effectiveness of milk feeding frequency (once or twice per day) using whole milk or milk replacer with an average TS content of 13% (Kehoe et al., 2007; Kienitz et al., 2017; Saldana et al., 2019). However, to our knowledge, there is no information on whether performance of young calf could be improved by feeding a wet starter diet when a high-solids MMR blend is offered once versus twice per day. The first hypothesis was that feeding frequency (twice versus once/d) would have a negligible effect on overall calf performance if calves receive an equivalent amount of nutrients from the high-solids MMR blend. Second, we hypothesized that feeding a moist starter diet would increase nutrient intake from the starter diet, thereby improving calf performance. Our main objective was to test the feasibility of developing a once-daily milk feeding program based on a high-solids MMR blend and a wet starter feed to promote calf performance and reduce labor costs.

The rearing and management programs and sampling procedures used throughout the experiment were approved by the Animal Care and Use Committee (IACUC #2020/005) described by the Iranian Council of Animal Care (1995).

A detailed description of calf management has been reported previously (Abbaslou et al., 2020; Norouzi et al., 2021). Forty-eight Holstein calves (24 male and 24 female; 40.2 ± 3.33 kg of BW) were recruited for the experiment, which was conducted on a commercial farm (Chaltasian Agri.-Animal Production Complex, Varamin, Iran). The experiment was conducted from June to September 2020. The temperature (outside barn) averaged 29.5°C during the experiment. Within the first 4 h of their life, calves received 4 L of high-quality colostrum (IgG concentration 50 mg/mL; Volac colostrometer). Individual calves were housed in pens 1.5 × 2.5 m in size. Calves were randomly assigned to 1 of 4 treatments. Treatments were a combination of 2 MMR feeding frequencies (once and twice daily) and 2 starter DM levels (91.1 and 50% DM), and included (1) once daily + dry starter feed (**OD**), (2) once daily + wet starter feed (**OW**), (3) twice daily + dry starter feed (TD), and (4) twice daily + wet starter feed (**TW**). The high-solids MMR blend was prepared by adding a calculated amount of the milk replacer powder to whole milk to ensure each program provides calves with a similar amount of nutrients from the MMR blend. The amount of milk replacer powder added to whole milk was adjusted according to the TS content of whole milk, which was analyzed regularly. The osmolality of whole milk and the MMR blend was determined using an osmometer (Osmomat 030, Gonotec). In the twice-daily program, the MMR blend was administered at a mean TS content of 18.6% (osmolality = 449 mOsm/kg) at a rate of 5 L/d from d 3 to d 55 (at 0900 and 1600 h in equivalent amounts) and 2.5 L/d at morning feeding on d 56 to weaning (d 60). In the once-daily program, the MMR blend with a mean TS content of 31.0% (osmolality = 650 mOsm/kg) was offered at a rate of 3 L/d of milk from d 3 to 55 and 1.5 L/d at morning feeding (at 0900) from d 56 to weaning (d 60). The milk replacer powder was a commercial product (Imperial, Novin Roshd Shahran Foudeh Co.), with the following basic ingredients: whole milk powder, skim milk powder, whey powder, whey protein concentrate, fats of plant origin, and mineral and vitamin supplement. Lactose, protein, fat, ash, and sodium concentrations (% of DM) in the milk replacer powder averaged 42.0, 21.9, 17.1, 8.11, and 1.06, respectively. Whole milk samples were analyzed weekly for protein (3.11%), fat (3.54%), lactose (4.49%), and TS (11.9%) using a CombiScope FTIR 600 Dairy analyzer (Delta Instruments). The osmolality of whole milk averaged 306 mOsm/kg. The preweaning period lasted until d 60 of calf life and the experiment was continued 30 d after weaning.

Calves had free access to water through a nipple. The starter diet was based on NRC (2001) recommendations and consisted of 83.5% ground ingredients (soybean meal, corn grain, and wheat bran) and 10% chopped wheat straw with a mean geometric particle size of 2.60 mm, as already described in our previous publication (Abbaslou et al., 2020). Sodium bicarbonate, calcium carbonate, salt, bentonite, and a vitamin and mineral mixture formed the remaining ingredients of the starter feed. The analyzed chemical composition (% of DM) of the starter feed including CP, fat, and NDF concentrations averaged 18.8, 2.72, and 24.3, respectively. The calculated ME content of the starter feed was 2.52 Mcal/kg of DM. The only difference between the starter feeds was their DM content. The calculated amount of water needed to reduce the dry starter's DM content to 50% was added to the dry starter daily in the morning in an electric mixer and thoroughly mixed for about 10 min. The starter feed was freely available throughout and its consumption was monitored daily. Calf weight was measured at 15-d intervals. During the milk feeding period, fecal consistency was individually assessed daily using the 5-point scale of Heinrichs et al. (2003).

For the primary response variables, including starter intake, BW, and ADG, a power analysis was conducted to estimate sample size (Morris, 1999; Hintze, 2008) based on previously published values (Miller-Cushon and DeVries, 2011). From the power test analysis with α = 0.05 and power = 0.80, the predicted sample size was 12 calves per treatment for starter DMI, ADG, and BW. Data were analyzed as a completely randomized design. Tukey's test was used for separation of the least squares means. Before analysis, all data were checked for normality using the UNIVARIATE Proc from SAS (SAS 9.4, SAS Institute Inc.). Data were subjected to ANOVA using the MIXED Proc of SAS with time as repeated measures for starter intake, ADG, and gain-to-feed ratio with the individual calf as the experimental unit. The model used calf birth weight as a covariate. Three variance-covariance structures (autoregressive type 1, compound symmetry, and Toeplitz) were tested, and autoregressive type 1 was selected based on Schwarz's minimized Bayesian information criterion. GENMOD Proc with a Poisson distribution was used to determine the difference between treatments in days with diarrhea, which was defined as fecal score ≥3 (Ahmadi et al., 2021). The significance threshold was set at *P* ≤ 0.05; trends were reported at *P* ≤ 0.10.

No calf mortality occurred during the experiment. The calves refused to drink the MMR blend during the first 10 d of life: an average of 0.22 and 0.08 L/d when fed once and twice per day, respectively. This resulted in an average DM consumption of high-solids MMR blend (d 3 to 60) of 0.89 g of DM/d in TD and TW calves compared with 0.87 g of DM/d in OD and OW calves (*P* = 0.01; Table 1).

**Table 1:** Effects of experimental treatments on performance of Holstein calves

Item	Treatment^1^				SEM	*P*-value
	OD	OW	TD	TW		
BW, kg						
Initial, d 3	40.2	40.2	40.5	39.9	0.43	0.96
Weaning, d 60	84.2	82.2	88.0	89.8	1.10	0.06
Final, d 90	119.8	114.8	124.0	123.3	1.46	0.09
Starter DMI, kg/d						
Preweaning, d 3 to 60	0.39	0.40	0.35	0.42	0.03	0.79
Postweaning, d 61 to 90	3.05^b^	3.22^ab^	3.06^b^	3.33^a^	0.06	0.05
Total, d 3 to 90	1.28	1.34	1.25	1.38	0.08	0.11
Milk DMI,^2^ kg/d						
Preweaning, d 3 to 60	0.870^b^	0.867^b^	0.886^a^	0.888^a^	0.01	0.01
Daily gain, kg/d						
Preweaning, d 3 to 60	0.73^bc^	0.70^c^	0.79^ab^	0.83^a^	0.02	<0.01
Postweaning, d 61 to 90	1.19	1.09	1.20	1.12	0.05	0.59
Total, d 3 to 90	0.88^ab^	0.83^b^	0.93^a^	0.93^a^	0.02	0.03
Total weight gain (kg), d 3 to 90	79.7^ab^	74.5^b^	83.5^a^	83.4^a^	1.28	0.04
Gain-to-feed ratio^3^						
Preweaning, d 3 to 60	0.60^b^	0.56^b^	0.67^a^	0.66^a^	0.01	<0.01
Postweaning, d 61 to 90	0.40	0.35	0.41	0.34	0.01	0.14
Total, d 3 to 90	0.53^ab^	0.49^b^	0.57^a^	0.55^a^	0.01	<0.01

a–c Within a row, least squares means with different superscripts differ (*P* ≤ 0.05).

1 Treatments were a combination of 2 milk and milk replacer (MMR) feeding frequencies (once versus twice daily) and 2 starter DM levels (91.1 and 50% DM), and included (1) once daily + dry starter diet (OD), (2) once daily + wet starter diet (OW), (3) twice daily + dry starter diet (TD), and (4) twice daily + wet starter diet (TW).

2 Dry matter intake from the high-solids MMR blend (whole milk + milk replacer powder).

3 kg of BW/DMI.

The effects of treatments based on the combination of feeding frequency of the MMR blend and DM content of starter feed on calf performance are shown in Table 1. Preweaning starter DMI was not different among treatments and averaged 0.39 kg of DM/d. However, in the postweaning period, a wet starter diet promoted starter feed intake: an average of 3.28 kg of DM/d for OW and TW versus 3.06 kg of DM/d for OD and TD calves. Feeding the high-solids MMR blend twice versus once daily generally promoted a greater calf growth rate, which was reflected in greater weaning weight (*P* = 0.06) in TD and TW (88.9 kg) than in OD and OW calves (83.2 kg). Final BW also followed the same trend and tended (*P* = 0.09) to be greater in calves that had received MMR blend twice a day during the milk feeding period. The daily growth rate after weaning did not differ among treatments and averaged 1.15 kg/d. The preweaning gain-to-feed ratio was greater in the TD and TW groups (*P* < 0.01) than in the OD and OW groups (mean 0.67 versus 0.58), but this difference disappeared after weaning and averaged 0.38 (*P* = 0.14). Throughout the whole period (d 3 to 90), TD and TW calves converted dietary nutrients into BW gains more efficiently than OW calves (0.56 vs. 0.49). Experimental treatments had no effects on days to first diarrhea (7.63 ± 0.49), duration of diarrhea (4.66 ± 0.38), or days treated with antibiotics (5.37 ± 0.36; Table 2).

**Table 2:** Effects of experimental treatments on diarrhea prevalence and days treated with antibiotics in Holstein calves

Item	Treatment^1^				SEM	*P*-value
	OD	OW	TD	TW		
Onset of diarrhea, d	7.58	7.91	7.31	7.71	0.49	0.86
Duration of diarrhea, d	5.00	4.83	4.80	4.00	0.38	0.61
Days treated with antibiotics^2^	5.17	5.09	6.10	5.10	0.36	0.39

1 Treatments were a combination of 2 milk and milk replacer feeding frequencies (once versus twice daily) and 2 starter DM levels (91.1 and 50% DM), and included (1) once daily + dry starter diet (OD), (2) once daily + wet starter diet (OW), (3) twice daily + dry starter diet (TD), and (4) twice daily + wet starter diet (TW).

2 The following antibiotics were used in accordance with the established veterinarian protocol: sodium sulfadiazine + trimethoprim (Trisul tablet, Zagros Pharmed Pars Co.), enrofloxacin (Enrocin, Razak Laboratories Co.), 1% neomycin (ScourSTOP, Livestock Drugs Production Co.), 20% apramycin (Apramax, Rooyan Darou Co.), and florfenicol (Razak Laboratories Co.).

Growth rate of calves fed the high-solids MMR blend once daily (OD and OW) averaged 715 g/d before weaning. This rate is comparatively greater than the mean values obtained in our previous trials with dairy calves under the comparable management systems, where whole milk with a mean solids content of about 11% was offered twice daily (Beiranvand et al., 2016, 2019; Fouladgar et al., 2016; Kargar et al., 2018). For example, Kargar et al. (2018) reported a mean growth rate before weaning (63 d) of 580 g/d when calves received a mean daily intake of 650 g of DM/d from whole milk (twice/d). This comparison suggests that a once-daily milk feeding strategy based on a high-solids MMR blend supports a potentially greater growth rate for calves than the conventional milk feeding systems based on whole milk twice daily. The greater nutrient intake from the liquid diet in the current trial compared with previous studies may have allowed the nutrient requirements for a faster growth rate to double the BW of the calves from birth to weaning. Calves showed no difference in their growth rate after weaning with an average of 1.15 ± 0.05 kg/d, which is close to the mean values (1.06–1.22 kg/d; from d 50 to 70) obtained in our previous works with dairy calves fed whole milk (TS = 10.8–11.0%; Beiranvand et al., 2016, 2019).

In calves receiving hypertonic solutions (600 mmol/L or higher), a delay in abomasal emptying regulates the flow of nutrients into the small intestine (Smith and Berchtold, 2014). Research has shown that feeding hypertonic fluids to calves results in a delay in abomasal emptying, which negatively affects calf growth (Burgstaller et al., 2017). The high-solids MMR blend was able to double the birth weight of calves until weaning when fed twice or once daily. This shows the feasibility of developing milk feeding strategies using high-solid liquids, especially with a once-daily feeding strategy, which offers some economic advantages.

Despite our initial hypothesis that a moist starter diet would promote starter intake and growth rate, such effects were not observed in this study. Our previous study with dairy calves fed a moist starter diet (DM = 50%) showed an average intake of 0.83 kg of starter DM/d before weaning (3 to 50 d; Beiranvand et al., 2016), which is significantly higher than the average values recorded in this trial (0.41 kg of DM/d; 3 to 60 d). This observation can be explained by the obtainment of the majority of calf daily nutrients from the high-solids MMR blend, which thus did not stimulate the calves to meet their nutrient requirements from the starter feed. In addition, increased nutrient intake from the high-solids MMR blend during the preweaning period could delay rumen development, which reduces nutrient digestibility and starter feed intake (Jasper and Weary, 2002; Hill et al., 2016). Khan et al. (2011) suggested that low intake of starters in studies with large amounts of whole milk or milk replacer is associated with satiety signals resulting from an increase in blood glucose and insulin, as well as gut filling associated with curd formation (Forbes, 1971).

It should be noted that this experiment was conducted in a dry environment and we detected no visible mold growth in the wet starter that was prepared on a daily basis. However, the addition of water to a starter feed in a humid environment and the assessment of mold growth in the starter must be further investigated.

Our previous studies reported that gain-to-feed ratio before weaning in Holstein calves fed whole milk (mean total DM = 11.4%) ranged from 0.46 to 0.53 (Beiranvand et al., 2016; Azad-Shahraki et al., 2019; Beiranvand et al., 2019). This indicates that the preweaning calves in this experiment were more efficient in converting nutrients into body gains. This result was expected because the substantially greater digestibility of nutrients in the MMR blend compared with the calf starter feed contributes to better gain-to-feed ratio in calves fed the high-solids MMR blend compared with whole milk. Calves fed whole milk rely more heavily on starter feed to meet their nutrient requirements for growth (Dennis et al., 2018).

There is a positive relationship between calf starter intake before weaning and growth rate after weaning (Stamey et al., 2012). This highlights the importance of management strategies that promote starter intake before weaning to maximize the potential of high-solids milk programs in calf-rearing systems. Mirzaei et al. (2020) reported that a step-down weaning strategy based on a milk solids intake of 912 g/d until 4 wk of age and then halving that amount (456 g/d) from d 29 to 63 (weaning) maximized starter milk intake during the preweaning period and reduced the negative effects of weaning stress on postweaning growth rate. Further research on such strategies would provide useful information on the interactions between starter DM content and feeding frequency of high-solids MMR blend on calf performance.

The increased osmolality of milk due to the addition of milk or milk replacer powders increases intestinal osmotic pressure and impairs intestinal barrier function, putting neonates at risk of osmotic diarrhea (Wilms et al., 2019). Although no specific threshold has been reported in the literature, caution should be exercised when administering liquid feeds with an osmolality greater than 600 mOsm/L (McGuirk, 2003). Contrary to our expectations, such effects were not observed in this experiment. Wilms et al. (2019) increased osmolality of milk replacer (from 439 to 611 mOsm/kg) through exchanging lactose with dextrose and confirmed that the calves did not exhibit serious health problems despite the impairment of gastrointestinal barrier function by the high-solids MMR feeding during their experiment. Similarly, Azevedo et al. (2016) found no difference in calf fecal consistency when the solids content of the MMR blend (whole milk + milk replacer powder) was increased from 13.5 to 20.4%, corresponding to an increase from 265 to 533 mOsm/L. It should also be noted that a liquid diet with an equivalent DM concentration consisting solely of milk replacer powder (not blended with milk) could have a different osmolality than the MMR blend. Calves may respond differently when fed the same concentration of solids from the milk replacer alone compared with the MMR blend, which warrants further investigation.

Under the conditions of the commercial farm where this experiment was conducted (milk feeding of approximately 400 calves per day), feeding the MMR blend once per day versus twice per day saved an estimated 3 h per day. This equates to daily savings of $0.09 per calf in labor costs (at $12 per labor hour) and labor savings of 0.45 min per calf. Implementing a once-a-day feeding strategy based on a high-solids MMR blend that requires less labor while avoiding a significant decline in overall calf performance could be especially important in emergency situations such as the emergence of COVID-19 pandemic that resulted in a serious shortage of labor in the agricultural workforce.

Under the conditions of this study, although the once-daily milk feeding program based on a high-solids MMR blend resulted in a reduction in calf growth rate before weaning, it resulted in calves weighing twice their birth weight at weaning. Contrary to our initial hypothesis, the addition of water to a dry fine-particle starter diet had no effect on starter intake before weaning, possibly due to increased nutrient intake from the high-solids MMR blend. Although calves preferred the wet over the dry starter after weaning, their growth rate did not differ, resulting in a greater gain-to-feed ratio for calves fed the dry starter. Feeding the high-solids MMR blend once daily as a less labor-intensive program could be a preferred management option in commercial calf-rearing systems and contribute to increased economic returns.
